# Clinical features and genetic characteristics of homozygous spinocerebellar ataxia type 3

**DOI:** 10.1002/mgg3.1314

**Published:** 2020-07-09

**Authors:** Quan-Fu Li, Hao‐Ling Cheng, Lu Yang, Yin Ma, Jing‐Jing Zhao, Yi Dong, Zhi‐Ying Wu

**Affiliations:** ^1^ Department of Neurology and Research Center of Neurology in Second Affiliated Hospital, and Key Laboratory of Medical Neurobiology of Zhejiang Province Zhejiang University School of Medicine Hangzhou China; ^2^ Department of Neurology and Institute of Neurology First Affiliated Hospital of Fujian Medical University Fuzhou China

**Keywords:** clinical features, gene dosage, homozygous, spinocerebellar ataxia type 3

## Abstract

**Background:**

Homozygous spinocerebellar ataxia type 3 (SCA3) patients, which have an expanded cytosine‐adenine‐guanine (CAG) repeat mutation in both alleles of *ATXN3*, are extremely rare. Clinical features and genetic characteristics of them were seldom studied.

**Methods:**

We analyzed seven newly homozygous SCA3 patients from five families and 14 homozygotes reported previously. An additional cohort of 30 heterozygous SCA3 patients were analyzed to compare age at onset (AAO).

**Results:**

Two out of seven SCA3 homozygotes had the minimum CAG repeats reported so far (55/56 and 56/58). Five patients appeared peripheral neuropathy and two had mild cognitive impairment. The AAO was significantly inversely correlated with both the large and small expanded CAG repeats (*r* = −.7682, *p* < .0001). The AAO was significantly earlier in homozygous SCA3 than heterozygous ones (32.81 ± 11.86 versus. 49.90 ± 9.73, *p* < .0001). In addition, the AAO of our seven homozygotes is elder compared to those reported previously (41.29 years vs. 28.57 years), which may be related to the fewer CAG repeats in our seven patients.

**Conclusion:**

Gene dosage effect may play an important role in the AAO and severity of disease, and homozygosity for *ATXN3* enhances phenotypic severity. Our findings expand clinical features and genetic characteristics of homozygous SCA3 patients.

## Graphical abstract

We analyzed seven newly homozygous spinocerebellar ataxia type 3 patients from five families and 14 homozygotes reported previously. The AAO was significantly inversely correlated with both the large and small expanded CAG repeats (*r* = −.7682, *p* < .0001). Gene dosage effect may play an important role in the AAO and severity of disease, and homozygosity for ATXN3 enhances phenotypic severity.

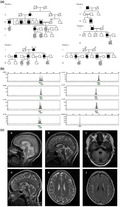

## 1 INTRODUCTION

Spinocerebellar ataxia type 3 (SCA3), an autosomal dominant cerebellar ataxia caused by abnormal expansion of cytosine‐adenine‐guanine (CAG) repeats in *ATXN3* gene (MIM: 109150) (Kawaguchi et al., 1994), is regarded as the most common SCA subtype in China (Gan et al., 2010). It was originally reported in a Portuguese‐Azorean family and named as Machado‐Joseph disease (Coutinho & Andrade, 1978). Clinical manifestations of SCA3 are heterogeneous, including cerebellar ataxia, external ophthalmoplegia, pyramidal and extrapyramidal signs, and peripheral neuropathy. Genetic anticipation phenomenon is seen in SCA3 patients. Age at onset (AAO) is inversely correlated with the size of CAG repeats in the pathogenic allele (Costa & Paulson, 2012). The polyglutamine (polyQ) diseases caused by CAG repeat expansions may share similar pathogenic mechanism. To study the genotype–phenotype relationship is of great help to understand the pathogenesis and develop rational, effective therapies for polyQ diseases.

Spinocerebellar ataxia type 3 is a relatively common subtype of polyQ diseases. While normal subjects have two normal alleles of *ATXN3* ranging between ten and 44 CAG repeats, SCA3 patients have at least one expanded allele ranging between 52 and 87 CAG repeats (Gan, Ni, Dong, Wang, & Wu, 2015). Since SCA3 is inherited in an autosomal dominant manner, homozygous SCA3 patients who have two expanded alleles of *ATXN3* are rarely reported. Prior to this report, a total of 14 homozygous SCA3 patients have been reported, including four Chinese patients (Carvalho, La Rocque‐Ferreira, Rizzo, Imamura, & Speck‐Martins, 2008; Fukutake et al., 2002; Lerer, Merims, Abeliovich, Zlotogora, & Gadoth, 1996; Lysenko, Grewal, Ma, & Peddareddygari, 2010; Shang et al., 2018; Sobue et al., 1996; Zeng et al., 2015). Compared with heterozygous SCA3, homozygous SCA3 has a double dosage effect on AAO and clinical phenotypes (Shang et al., 2018).

However, a comprehensive and systematic research on homozygous SCA3 is absent. The correlation between AAO and CAG repeats in homozygous SCA3 patients is unknown. Here we reported seven Chinese homozygous SCA3 patients and summarized all previously reported homozygous SCA3 patients. Our study provided additional genetic and clinical features of homozygous SCA3.

## 2 MATERIALS AND METHODS

### 2.1 Subjects

Seven homozygous SCA3 patients and 12 heterozygous SCA3 subjects from five families were recruited after genetic testing of *ATXN3* between February 2015 and February 2019. Another 30 unrelated heterozygous SCA3 patients were recruited as controls. The disease severity with the scale for the assessment and rating of ataxia (SARA, 0–40) and the international cooperative ataxia rating scale (ICARS, 0–100) was recorded. Cognitive status was assessed by Mini‐Mental State Examination (MMSE, 0–30). This study was approved by the Ethics Committee for clinical medical research and each participant gave a written informed consent.

### 2.2 Genetic analysis

Genomic DNA was extracted from venous blood samples from participants using Blood Genomic Extraction Kit (Qiagen). Genetic testing was conducted as previously reported (Kawaguchi et al., 1994). The CAG repeats number of heterozygous SCA3 was determined by polymerase chain reaction amplification combined with Sanger sequencing as described in our previous studies (Gan et al., 2010, 2015). The CAG repeats number of homozygous SCA3 was conducted using DNA fragment analysis by capillary electrophoresis on an ABI 3730XL DNA analyzer (XiangYin Biotechnology).

### 2.3 Statistical analysis

Statistical analyses including Pearson's correlation coefficient was performed using GraphPad Prism v7.0 for Windows (GraphPad Software, www.graphpad.com). Mann–Whitney test was used to compare groups. The threshold for statistical significance was *p* < .05.

## 3 RESULTS

### 3.1 Genetic findings

**Figure 1:**
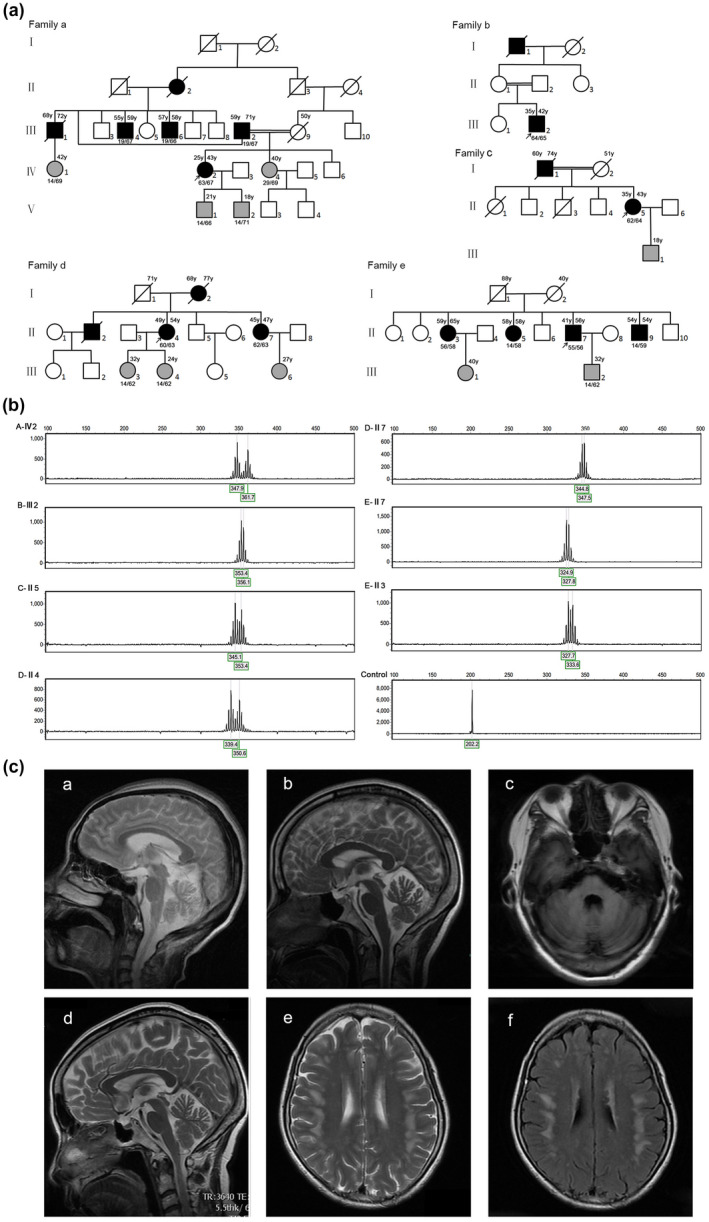
(A) The pedigree trees of the five homozygous SCA3 families (Family a‐e). The square indicates male, circle indicates female; dark fill represents the affected individual and gray fill indicates the carrier without symptom. The diagonal line indicates the deceased individual. Age at onset is given on the top left‐hand and the current age/death age on the top right‐hand. The number of CAG repeats of the *ATXN3* allele pair is given beneath symbols. (B) DNA fragment analysis by capillary electrophoresis. From family A‐IV2 to E‐II3: seven homozygous SCA3 cases. Control: standard sample (14 CAG repeats in the *ATXN3* allele). (C) Cranial magnetic resonance image (MRI) of the available homozygous SCA3 patients. a: mild cerebellar atrophy of Case 1 (Family A, IV2); b: cerebellar atrophy of Case 3 (Family C, II‐5); c: cerebellar atrophy of Case 3 (Family D, II‐4); d, e, f: cerebellum atrophy and demyelination in frontal and parietal lobes of Case 7 (Family E, II‐3). A, b, d: sagittal T2; c: axial T1; e: axial T2; f: axial Flair. SCA3, spinocerebellar ataxia type 3

Genetic testing in the five families (Figure 1a) revealed that seven patients had biallelic expansions in *ATXN3*. The expanded CAG repeats were 60.29 ± 3.50 (small, range 55–64) and 62.29 ± 3.90 (large, range 56–67), respectively (Figure 1b, Table 3). Of seven SCA3 homozygotes, two had an expanded CAG repeat less than 60 (55/56 and 56/58). Twelve heterozygous SCA3 individuals were identified in five families, including seven pre‐symptomatic (expanded CAG range 62–71) and five manifest patients (expanded CAG range 58–67). The expanded CAG repeats of 30 unrelated heterozygous SCA3 patients was 67.63 ± 3.50 (range 58–72) (Table 3).

### 3.2 Clinical features of homozygous SCA3 patients

The clinical features of seven homozygous SCA3 patients are summarized in Table 1. Three out of five families were consanguineous marriages. The mean AAO was 41.3 years and the oldest AAO was 59 years. All patients presented with symptoms of cerebellar ataxia, including unsteady gait, slurred speech, and dysphagia. The symptoms progressed gradually. Five out of seven (71.4%, 5/7) homozygous SCA3 patients presented with peripheral neuropathy.

**Table 1:** Clinical features of homozygous SCA3 patients in this study

Patient	Gender	Age at onset (years)	Duration (years)	CAG repeats	SARA	ICARS	Clinical features
Case 1	Female	25	18	63/67	32/40	57/100	Horizontal and vertical nystagmus, up‐gaze limitations, dysarthria, gait ataxia, peripheral neuropathy, cognitive impairment
Case 2	Male	35	7	64/65	NA	NA	Horizontal nystagmus and binocular abduction limitations, dysphagia, slurred speech, gait ataxia, peripheral neuropathy
Case 3	Female	35	8	62/64	15/40	42/100	Bulging eyes, nystagmus, dysarthria, dysphagia, constipation, urinary incontinence, peripheral neuropathy, depression
Case 4	Female	49	5	60/63	13/40	33/100	Nystagmus, dysarthria, gait ataxia, peripheral neuropathy
Case 5	Female	45	2	62/63	9/40	NA	Gait ataxia, dysarthria, horizontal nystagmus
Case 6	Male	41	15	55/56	NA	20/100	Gait ataxia
Case 7	Female	59	6	56/58	5/40	14/100	Gaze‐evoked horizontal nystagmus, gait ataxia, hyperactive tendon reflexes, cramps and cold feeling in the legs

Abbreviations: ICARS, international cooperative ataxia rating scale; NA, not available; SARA, scale for the assessment and rating of ataxia; SCA3, spinocerebellar ataxia type 3.

Case 1 (Family a, IV‐2) is a female patient who suffered dizzy with slow progression of gait unsteadiness and dysarthria at the age of 25. Family history disclosed that the parents are first cousins. She needs a mobility aid to protect the safety of walking when she was 33 years old. She gradually developed double vision and dysphagia. At the age of 35, she was evaluated at the outpatient clinic of our hospital for the first time. Brain magnetic resonance imaging (MRI) showed mild cerebellar and brainstem atrophy. Three years later, the patient began to fall frequently and had to use wheelchair. During the recent 4 years, she suffered from facial hypomimia with open mouth and drooling, constipation, and sleep disorders. Neurological examinations revealed severe truncal and limb ataxia. The extraocular movements showed gaze‐evoked vertical and horizontal nystagmus and up‐gaze limitations. Tendon areflexia, bilateral extensor plantar reflex, and muscular atrophy of both lower limbs were noted. Decreased vibration sense was observed, but position sense was normal. She had slight cognitive dysfunction (MMSE 16/30, primary school education level). The clinical scales of SARA and ICARS were evaluated (SARA = 32/40, ICARS = 57/100). Brain MRI was performed again and cerebellar atrophy aggravated slightly (Figure 1c‐a). An electrophysiological study showed evidence of sensory and motor peripheral neuropathy. Her father (III‐2) developed gait disturbance at age of 59 and double vision at age of 65. She is 71 years old now and had no dysarthria and dysphagia. Her mother (III‐9) was asymptomatic and died of endometrial carcinoma at age of 50. In addition, the patient's uncles (III‐1, III‐4, and III‐6) appeared slightly unsteady gait after 55 years old. Her cousins had no clinical symptoms up to the present age (IV‐1, 42 years old, IV‐4, 40 years old).

Case 2 (Family b, III‐2) is a 37‐year‐old man with a 2‐year history of cerebellar ataxia including unsteady walk, dysphagia, and slurred speech. He is a child of first‐cousin consanguineous parents. His parents had no gait problem, but detailed neurological assessment was not done. Upon neurological examinations, significant nystagmus and binocular abduction limitations were observed. Distinctly abnormal finger‐nose test and heel‐knee‐tibia test were noted. Pyramidal and extrapyramidal signs, muscle weakness or paresthesia were not observed. Brain MRI showed cerebellar atrophy. An electrophysiological study revealed mainly decreased sensory nerve conduction velocity, supporting the multiple peripheral neuropathy.

Case 3 (Family c, II‐5) is a female patient who developed gait problem at her 35 years old. Brain MRI showed mild cerebellar atrophy at her 38 years old (Figure 1c‐b). Her clinical symptoms worsened quickly when she was 43 years old. She fell frequently and developed severe dizzy especially at horizontal position. Severe dysarthria and dysphagia were observed. She reported a feeling of cold in both legs. She experienced problems of constipation and urinary incontinence. Neurological examinations revealed mild bulging eyes, frequent blinking, nystagmus, paresthesia of both lower extremities. The clinical scales showed a severe disease stage (SARA = 15/40, ICARS = 42/100). She was anxious about her illness and suffered from mild anxiety and depression. She has thoughts of hurting herself or committing suicide. The family history revealed that her father had developed unsteadiness of gait in the sixth decade of life and died at age of 74 for unknown reason. Her mother died of liver cancer at age of 51 without any cerebellar symptoms.

Case 4 (Family D, II‐4) developed dizzy and body sway at age of 49. These symptoms gradually progressed and slurred speech appeared at age of 52. She complained of anxiety and depression. Neurological examinations revealed moderate nystagmus. Tendon reflex of upper limbs was normal, but deep tendon reflex of both lower limbs was absent. An electrophysiological study showed mild decreased conduction velocity of both motor and sensory nerves. Brain MRI showed mild cerebellum atrophy (Figure 1c‐c). Her father died at age of 71 without cerebellar ataxia. Her mother walked unsteadily at age of 68 and died of cerebral hemorrhage at age of 77. Her young sister (Case 5, II‐7) had mild gait ataxia at her 47 years old. No diplopia, dysphagia, and slurred speech symptoms were observed. Neurological exam revealed showed gaze‐evoked horizontal nystagmus. Her elder brother (II‐2) appeared similar symptoms in his 30 s, and died at 49 years old for unknown reasons. Her two daughters still didn't have any ataxia symptoms and neurological exam was normal.

Case 6 (Family e, II‐7) appeared unsteady gait at age of 41. The symptoms were slightly progressed. His speech is fluent and vision is normal. No abnormal neurological examination was observed except mild limbs ataxia. His elder sister (case 7, II‐3) was also a homozygous SCA3 patient. She developed head dizzy and clumsy walking when she was 59 years old. At 64 years old, she complained of cramps in the legs and cold feeling on the soles of the feet. Neurological examinations showed gaze‐evoked horizontal nystagmus and brisk tendon reflexes of both lower extremities. She had mild cognitive impairment (MMSE 17/30, illiterate). Her activity of daily living scale was normal. Cranial MRI demonstrated cerebellum atrophy and demyelination in both frontal and parietal lobes (Figure 1c,d‐f).

### 3.3 Correlation between CAG repeats and AAO of homozygous SCA3

**Figure 2:**
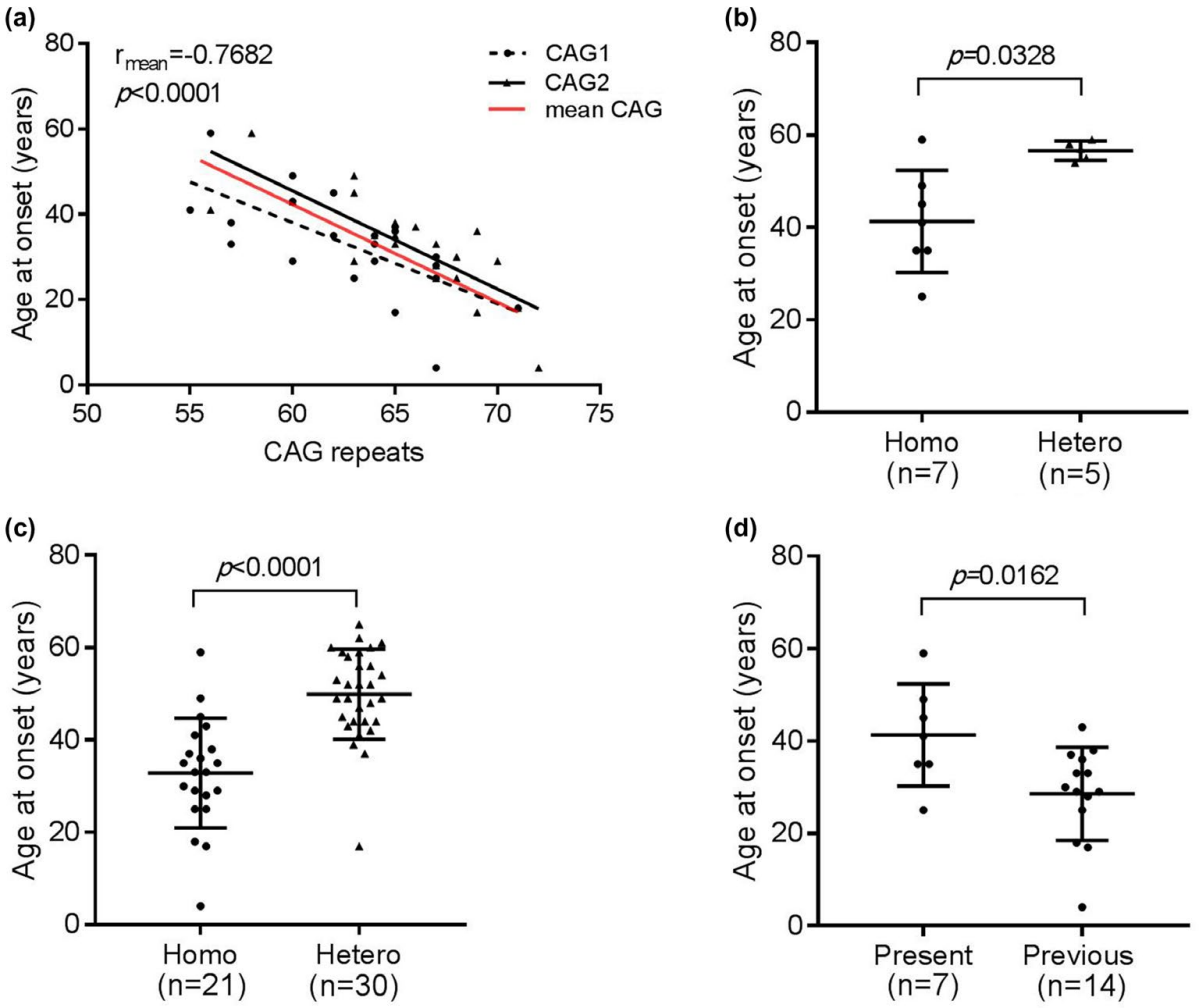
Comparison of age at onset between homozygous and heterozygous SCA3 patients. (a) Age at onset is inversely correlated to CAG repeats of small (CAG1) and large (CAG2) expanded allele in 21 homozygous SCA3 patients. The correlation coefficient of age at onset and mean CAG repeats is −0.7682 (Pearson *r*, *p* < .0001). (b) Age at onset is earlier in homozygous SCA3 patients (*n* = 7) than heterozygous SCA3 patients (*n* = 5) from present five families. (c) Age at onset is earlier in all homozygous SCA3 patients (*n* = 21) than heterozygous SCA3 patients (*n* = 30). (d) Age at onset of homozygous SCA3 patients is elder in present study (*n* = 7) than in previous studies (*n* = 14). SCA3, spinocerebellar ataxia type 3

We collected the clinical and genetic information of 14 homozygous SCA3 patients from previous reports^6‐12^. Adding our seven homozygous SCA3 patients, the mean AAO of all 21 homozygous SCA3 patients was 32.8 years old (Table 2). After the linear regression analysis, we found that AAO was significantly inversely correlated with both the large (CAG1) and small (CAG2) expanded CAG repeats, and the mean correlation coefficient was *r* = −.7682 (*p* < .0001, Figure 2a). As shown in Table 3, a comparison of AAO between homozygous SCA3 and heterozygous SCA3 patients was studied in our five families. No significance of larger expanded CAG repeats was seen. However, AAO of homozygous SCA3 was earlier than that of heterozygous SCA3 (41.29 ± 11.04 vs. 56.6 ± 2.07, *p* = .0328) (Figure 2b). Next, we compared AAO in all 21 homozygous SCA3 patients and independent 30 heterozygous SCA3 patients. More significant difference of AAO was observed (32.81 ± 11.86 versus. 49.90 ± 9.73, *p* < .0001, Figure 2c). Besides, we found that the AAO of our seven homozygous SCA3 patients was elder compared to the 14 previously reported patients (41.29 ± 11.04 vs. 28.57 ± 10.10 years, *p* = .0162) (Figure 2d).

**Table 2:** Clinical features of homozygous 21 SCA3 patients

Patient	Race	Gender	AAO	CAG1	CAG2	References
1	Chinese	Female	25	63	67	Present study
2	Chinese	Male	35	64	65	Present study
3	Chinese	Female	35	62	64	Present study
4	Chinese	Female	49	60	63	Present study
5	Chinese	Female	45	62	63	Present study
6	Chinese	Male	41	55	56	Present study
7	Chinese	Female	59	56	58	Present study
8	Chinese	Male	33	64	67	Shang et al., 2018
9	Chinese	Female	38	57	65	Shang et al., 2018
10	Chinese	Female	33	57	65	Shang et al., 2018
11	Chinese	Male	18	71	71	Zeng et al., 2015
12	Portuguese/Brazilian	Male	29	60	63	Lysenko et al., 2010
13	Japanese	Male	43	60	60	Fukutake et al., 2002
14	Brazilian	Female	4	67	72	Carvalho et al., 2008
15	Japanese	Male	28	67	67	Sobue et al., 1996
16	Yemenite Jewish	NA	29	64	70	Lerer et al., 1996
17	Yemenite Jewish	NA	37	65	66	Lerer et al., 1996
18	Yemenite Jewish	NA	25	67	68	Lerer et al., 1996
19	Yemenite Jewish	NA	30	67	68	Lerer et al., 1996
20	Yemenite Jewish	NA	17	65	69	Lerer et al., 1996
21	Yemenite Jewish	NA	36	65	69	Lerer et al., 1996
Total	/	/	32.8 (4–59)	62.8 (55–71)	65.5 (56–72)	/

Abbreviations: “/”, not applicable; AAO, age at onset; CAG1, CAG repeats of small expansion allele; CAG2, CAG repeats of larger expansion allele; NA, not available; SCA3, spinocerebellar ataxia type 3.

**Table 3:** Demographics of SCA3 homozygotes and heterozygotes

	Group 1		Group 2	
	Homozygotes (*n* = 7)	Heterozygotes (*n* = 5)	Homozygotes (*n* = 21)	Heterozygotes (*n* = 30)
Male/Female	2:5	4:1	7:8 ^a^	14:16
Age at onset, y	41.29 ± 11.04	56.6 ± 2.07	32.81 ± 11.86	49.90 ± 9.73
Age at examination, y	50.00 ± 8.64	60.00 ± 6.44	43.07 ± 13.66 ^a^	55.23 ± 11.20
Disease duration, y	8.71 ± 5.71	3.80 ± 4.76	8.73 ± 4.94 ^a^	7.40 ± 4.29
Large CAG repeat	62.29 ± 3.90	63.40 ± 4.51	65.67 ± 3.95	67.63 ± 3.50
Small CAG repeat	60.29 ± 3.50	17.00 ± 2.74	62.76 ± 4.22	18.63 ± 7.09

Note

Values are given as mean ± *SD*. Group 1: SCA3 patients from five families of the present study. Group 2: all homozygous SCA3 patients and 30 independent heterozygous SCA3 patients. “a”: data from six patients (reference Lerer et al., 1996) were not available, only 15 patients were analyzed.

Abbreviation: SCA3, spinocerebellar ataxia type 3.

## 4 DISCUSSION

A strong negative correlation between size of CAG repeats and AAO has been reported in heterozygous SCA3 patients (Chen et al., 2016; Donis et al., 2016; Globas et al., 2008; Netravathi et al., 2009). The higher the size of CAG repeats, the earlier the onset age. The AAO of SCA3 patients is mainly determined by the expanded CAG repeats, approximately 45%–87% of the total AAO variance of SCA3 (Maciel et al., 1995; van de Warrenburg et al., 2002, 2005). However, the relationship between size of CAG repeats and AAO in homozygous SCA3 patients remains unknown, since homozygous SCA3 patients are very rare.

In this study, we reported seven newly homozygous SCA3 patients (the largest cohort so far) and summarized 14 previously reported SCA3 homozygotes. Two of our patients had CAG repeats less than 60 in both alleles (55/56 and 56/58), which were the smallest size of CAG repeats reported in homozygous SCA3 patients. Unfortunately, six of our seven homozygous SCA3 patients have already given birth to children, all of whom are carriers of mutant *ATXN3*. Consanguineous marriage should be avoided and it is important to block the transmission of mutant *ATXN3* to the next generation by genetic counselling.

In addition, we first described the correlation between size of CAG repeats and AAO in totally 21 homozygous SCA3 patients. The length of expanded CAG repeats can determine 59% of AAO variance in homozygous SCA3, which is similar to that of Chinese heterozygous SCA3 patients (63%) (Chen et al., 2016). However, the AAO of 21 homozygous SCA3 patients varied greatly, range from 4 to 59 years old. We observed that patients with similar expanded CAG repeats had significantly different AAO, suggesting other factors modulating AAO. Several other factors are responsible for the residual variance. A previous study demonstrated familial factors modulate AAO of SCA3 (DeStefano et al., 1996). Methylation levels in the promotor region of *ATXN3* were detected with earlier AAO and influenced the instability of intergenerational CAG repeats (Wang et al., 2017). A study reported the *APOE* alleles might be genetic modifiers for AAO in SCA3 (Peng et al., 2014). However, our previous research did not support the role of *APOE* in a large cohort of SCA3 patients (Zhou et al., 2014). Besides, mitochondrial NADH dehydrogenase subunit 3 polymorphism might be associated with an earlier AAO (Chen et al., 2016). So far, the mechanism underlying this observation is still not completely understood and much more research is needed.

As we known, *ATXN3* is a deubiquitinating enzyme, which plays a role in ubiquitin‐mediated proteolysis and in the regulation of the ubiquitination status. Mutant *ATXN3* appears to be dysfunctional by forming insoluble aggregates primarily in the nucleus and interacting abnormally with other protein partners (Klockgether, Mariotti, & Paulson, 2019). In the current study, we demonstrated that AAO was significantly earlier in homozygous SCA3 than heterozygous SCA3. Although the pathogenesis of earlier onset in homozygous SCA3 is still unknown, gene dosage effect may play an important role in the AAO and severity of disease. We found that the AAO of homozygous SCA3 patients was negatively correlated with CAG repeats of both large and small expanded alleles. In addition, the AAO of our seven homozygous SCA3 patients is elder compared to those reported previously (41.29 years vs. 28.57 years), which may be related to the fewer CAG repeats in our seven patients. Thus, the homozygous SCA3 patients could harbor only two much smaller alleles compared to heterozygous patients, otherwise, the dosage effect might lead to an early death or developmental malfunction so that patients could not be detected. Even the normal CAG repeat at *ATXN3* has a small but significant influence on AAO of SCA3 (Zhou et al., 2014), it is reasonable to assume that homozygosity enhances the clinical phenotypes through complete loss of normal function and increased toxic protein effects. In addition, homozygotes in other SCA subtypes, such as SCA2, SCA6, and SCA17, also appeared early onset and atypical symptoms than heterozygotes (Hire, Katrak, Vaidya, Radhakrishnan, & Seshadri, 2011; Kato et al., 2000; Ragothaman et al., 2004; Soga et al., 2017; Takahashi et al., 2004; Toyoshima et al., 2004; Zühlke et al., 2003).

Cranial MRI is an effective marker for clinical studies of SCA. Generally, the volume of cerebellar is negatively correlated with the clinical severity of SCA (Eichler et al., 2011). Interestingly, one of homozygous SCA3 patients (Family a, IV‐2) presented relatively reserved cerebellum and brain stem volume on MRI, while the clinical stage of this patient is already late stage (SARA 32/40). Similar phenomenon existed in a previously reported case (Zeng et al., 2015) and the exact pathological mechanism is complicated and still unclear. SCA3 patients presented with severe atrophy throughout the total brainstem (midbrain, pons, and medulla), the entire cerebellum, putamen and caudate nucleus (Schulz et al., 2010). Demyelination in both frontal and parietal lobes were observed in one of SCA3 homozygotes (Family e, II‐7), which was firstly reported in the homozygous SCA3 patient. Cognitive decline may be related to demyelination of cerebrum. Besides, we did electrophysiological study in three homozygous SCA3 patients and all appeared peripheral neuropathy changes. Two out of the left four cases existed abnormal sensory feeling in limbs. In all 21 homozygous SCA3 patients, 8 patients (38.1%, 8/21) presented with peripheral neuropathy, while in our 30 heterozygous SCA3 patients, 5 patients (16.7%, 5/30) presented with peripheral neuropathy. Therefore, the SCA3 homozygotes may be more prone to peripheral neuropathy. This result needs to be validated in more homozygous SCA3 patients.

In summary, we presented seven homozygous SCA3 patients from five families and described clinical features of them. In addition, 14 previously reported SCA3 homozygotes were summarized and the correlation between size of CAG repeats and AAO was analyzed. This study expands clinical features and genetic characteristics of homozygous SCA3 patients.

## CONFLICT OF INTEREST

The authors declare that they have no conflict of interest.

## AUTHOR CONTRIBUTIONS

All authors gave approval for the final version of manuscript. Z‐Y.W. conceptualized the study. Y.D. and Z‐Y.W. performed physical examination of the patients and analyzed clinical data. Q‐F.L., H‐L.C., L.Y., Y. M., and J‐J. Z. contributed to acquisition of data. Q‐F.L., H‐L.C., Y.D., and Z‐Y.W. contributed to interpretation of data. Q‐F.L. drafted the manuscript and Z‐Y.W. critically revised the manuscript.

## ETHICS APPROVAL

The study was approved by the Ethics Committee for clinical medical research. Informed consents were obtained from participants included in the study or their guardians.

## Data Availability

The data used to support the findings of this study are available from the corresponding author upon request.

## References

[mgg31314-bib-0001] Carvalho, D. R. , La Rocque‐Ferreira, A. , Rizzo, I. M. , Imamura, E. U. , & Speck‐Martins, C. E. (2008). Homozygosity enhances severity in spinocerebellar ataxia type 3. Pediatric Neurology, 38(4), 296–299. 10.1016/j.pediatrneurol.2007.12.006 18358414

[mgg31314-bib-0002] Chen, S. , Gan, S.‐R. , Cai, P.‐P. , Ni, W. , Zhou, Q. I. , Dong, Y. I. , … Wu, Z.‐Y. (2016). Mitochondrial NADH dehydrogenase subunit 3 polymorphism associated with an earlier age at onset in male Machado‐Joseph disease patients. CNS Neuroscience & Therapeutics, 22(1), 38–42. 10.1111/cns.12443 26336829 PMC6492796

[mgg31314-bib-0003] Costa, M. C. , & Paulson, H. L. (2012). Toward understanding Machado‐Joseph disease. Progress in Neurobiology, 97(2), 239–257. 10.1016/j.pneurobio.2011.11.006 22133674 PMC3306771

[mgg31314-bib-0004] Coutinho, P. , & Andrade, C. (1978). Autosomal dominant system degeneration in Portuguese families of the Azores Islands. A new genetic disorder involving cerebellar, pyramidal, extrapyramidal and spinal cord motor functions. Neurology, 28(7), 703–709. 10.1212/wnl.28.7.703 566869

[mgg31314-bib-0005] DeStefano, A. L. , Cupples, L. A. , Maciel, P. , Gaspar, C. , Radvany, J. , Dawson, D. M. , … Et, A. (1996). A familial factor independent of CAG repeat length influences age at onset of Machado‐Joseph disease. American Journal of Human Genetics, 59(1), 119–127. 8659514 PMC1915115

[mgg31314-bib-0006] Donis, K. C. , Saute, J. A. M. , Krum‐Santos, A. C. , Furtado, G. V. , Mattos, E. P. , Saraiva‐Pereira, M. L. , … Jardim, L. B. (2016). Spinocerebellar ataxia type 3/Machado‐Joseph disease starting before adolescence. Neurogenetics, 17(2), 107–113. 10.1007/s10048-016-0473-5 26780339

[mgg31314-bib-0007] Eichler, L. , Bellenberg, B. , Hahn, H. K. , Koster, O. , Schols, L. , & Lukas, C. (2011). Quantitative assessment of brain stem and cerebellar atrophy in spinocerebellar ataxia types 3 and 6: Impact on clinical status. American Journal of Neuroradiology, 32(5), 890–897. 10.3174/ajnr.A2387 21372168 PMC7965570

[mgg31314-bib-0008] Fukutake, T. , Shinotoh, H. , Nishino, H. , Ichikawa, Y. , Goto, J. , Kanazawa, I. , & Hattori, T. (2002). Homozygous Machado‐Joseph disease presenting as REM sleep behaviour disorder and prominent psychiatric symptoms. European Journal of Neurology, 9(1), 97–100. 10.1046/j.1468-1331.2002.00335.x 11784384

[mgg31314-bib-0009] Gan, S. R. , Ni, W. , Dong, Y. , Wang, N. , & Wu, Z. Y. (2015). Population genetics and new insight into range of CAG repeats of spinocerebellar ataxia type 3 in the Han Chinese population. PLoS ONE, 10(8), e134405 10.1371/journal.pone.0134405 PMC4534407 26266536

[mgg31314-bib-0010] Gan, S.‐R. , Shi, S.‐S. , Wu, J.‐J. , Wang, N. , Zhao, G.‐X. , Weng, S.‐T. , … Wu, Z.‐Y. (2010). High frequency of Machado‐Joseph disease identified in southeastern Chinese kindreds with spinocerebellar ataxia. BMC Medical Genetics, 11, 47 10.1186/1471-2350-11-47 20334689 PMC2861663

[mgg31314-bib-0011] Globas, C. , du Montcel, S. T. , Baliko, L. , Boesch, S. , Depondt, C. , DiDonato, S. , … Schols, L. (2008). Early symptoms in spinocerebellar ataxia type 1, 2, 3, and 6. Movement Disorders, 23(15), 2232–2238. 10.1002/mds.22288 18759344

[mgg31314-bib-0012] Hire, R. R. , Katrak, S. M. , Vaidya, S. , Radhakrishnan, K. , & Seshadri, M. (2011). Spinocerebellar ataxia type 17 in Indian patients: Two rare cases of homozygous expansions. Clinical Genetics, 80(5), 472–477. 10.1111/j.1399-0004.2010.01589.x 21108634

[mgg31314-bib-0013] Kato, T. , Tanaka, F. , Yamamoto, M. , Yosida, E. , Indo, T. , Watanabe, H. , … Sobue, G. (2000). Sisters homozygous for the spinocerebellar ataxia type 6 (SCA6)/CACNA1A gene associated with different clinical phenotypes. Clinical Genetics, 58(1), 69–73. 10.1034/j.1399-0004.2000.580112.x 10945665

[mgg31314-bib-0014] Kawaguchi, Y. , Okamoto, T. , Taniwaki, M. , Aizawa, M. , Inoue, M. , Katayama, S. , … Kakizuka, A. (1994). CAG expansions in a novel gene for Machado‐Joseph disease at chromosome 14q32.1. Nature Genetics, 8(3), 221–228. 10.1038/ng1194-221 7874163

[mgg31314-bib-0015] Klockgether, T. , Mariotti, C. , & Paulson, H. L. (2019). Spinocerebellar ataxia. Nature Reviews Disease Primers, 5(1), 24 10.1038/s41572-019-0074-3 30975995

[mgg31314-bib-0016] Lerer, I. , Merims, D. , Abeliovich, D. , Zlotogora, J. , & Gadoth, N. (1996). Machado‐Joseph disease: Correlation between the clinical features, the CAG repeat length and homozygosity for the mutation. European Journal of Human Genetics, 4(1), 3–7. 10.1159/000472162 8800925

[mgg31314-bib-0017] Lysenko, L. , Grewal, R. P. , Ma, W. , & Peddareddygari, L. R. (2010). Homozygous Machado Joseph disease: A case report and review of literature. Canadian Journal of Neurological Sciences, 37(4), 521–523. 10.1017/s0317167100010581 20724264

[mgg31314-bib-0018] Maciel, P. , Gaspar, C. , DeStefano, A. L. , Silveira, I. , Coutinho, P. , Radvany, J. , … Et, A. (1995). Correlation between CAG repeat length and clinical features in Machado‐Joseph disease. American Journal of Human Genetics, 57(1), 54–61. 7611296 PMC1801255

[mgg31314-bib-0019] Netravathi, M. , Pal, P. K. , Purushottam, M. , Thennarasu, K. , Mukherjee, M. , & Jain, S. (2009). Spinocerebellar ataxias types 1, 2 and 3: Age adjusted clinical severity of disease at presentation correlates with size of CAG repeat lengths. Journal of the Neurological Sciences, 277(1–2), 83–86. 10.1016/j.jns.2008.10.016 19049837

[mgg31314-bib-0020] Peng, H. , Wang, C. , Chen, Z. , Sun, Z. , Jiao, B. , Li, K. , … Jiang, H. (2014). APOE epsilon2 allele may decrease the age at onset in patients with spinocerebellar ataxia type 3 or Machado‐Joseph disease from the Chinese Han population. Neurobiology of Aging, 35(9), 2115–2179. 10.1016/j.neurobiolaging.2014.03.020 24746364

[mgg31314-bib-0021] Ragothaman, M. , Sarangmath, N. , Chaudhary, S. , Khare, V. , Mittal, U. , Sharma, S. , … Muthane, U. B. (2004). Complex phenotypes in an Indian family with homozygous SCA2 mutations. Annals of Neurology, 55(1), 130–133. 10.1002/ana.10815 14705123

[mgg31314-bib-0022] Schulz, J. B. , Borkert, J. , Wolf, S. , Schmitz‐Hübsch, T. , Rakowicz, M. , Mariotti, C. , … Hauser, T.‐K. (2010). Visualization, quantification and correlation of brain atrophy with clinical symptoms in spinocerebellar ataxia types 1, 3 and 6. NeuroImage, 49(1), 158–168. 10.1016/j.neuroimage.2009.07.027 19631275

[mgg31314-bib-0023] Shang, X.‐J. , Xu, H.‐L. , Yang, J.‐S. , Chen, P.‐P. , Lin, M.‐T. , Qian, M.‐Z. , … Gan, S.‐R. (2018). Homozygote of spinocerebellar Ataxia type 3 correlating with severe phenotype based on analyses of clinical features. Journal of the Neurological Sciences, 390, 111–114. 10.1016/j.jns.2018.04.026 29801869

[mgg31314-bib-0024] Sobue, G. , Doyu, M. , Nakao, N. , Shimada, N. , Mitsuma, T. , Maruyama, H. , … Nakamura, S. (1996). Homozygosity for Machado‐Joseph disease gene enhances phenotypic severity. Journal of Neurology, Neurosurgery and Psychiatry, 60(3), 354–356. 10.1136/jnnp.60.3.354-a PMC1073875 8609529

[mgg31314-bib-0025] Soga, K. , Ishikawa, K. , Furuya, T. , Iida, T. , Yamada, T. , Ando, N. , … Yokota, T. (2017). Gene dosage effect in spinocerebellar ataxia type 6 homozygotes: A clinical and neuropathological study. Journal of the Neurological Sciences, 373, 321–328. 10.1016/j.jns.2016.12.051 28131213

[mgg31314-bib-0026] Takahashi, H. , Ishikawa, K. , Tsutsumi, T. , Fujigasaki, H. , Kawata, A. , Okiyama, R. , … Mizusawa, H. (2004). A clinical and genetic study in a large cohort of patients with spinocerebellar ataxia type 6. Journal of Human Genetics, 49(5), 256–264. 10.1007/s10038-004-0142-7 15362569

[mgg31314-bib-0027] Toyoshima, Y. , Yamada, M. , Onodera, O. , Shimohata, M. , Inenaga, C. , Fujita, N. , … Takahashi, H. (2004). SCA17 homozygote showing Huntington's disease‐like phenotype. Annals of Neurology, 55(2), 281–286. 10.1002/ana.10824 14755733

[mgg31314-bib-0028] van de Warrenburg, B. P. C. , Hendriks, H. , Dürr, A. , van Zuijlen, M. C. A. , Stevanin, G. , Camuzat, A. , … Kremer, B. P. H. (2005). Age at onset variance analysis in spinocerebellar ataxias: A study in a Dutch‐French cohort. Annals of Neurology, 57(4), 505–512. 10.1002/ana.20424 15747371

[mgg31314-bib-0029] van de Warrenburg, B. , Sinke, R. J. , Verschuuren‐Bemelmans, C. C. , Scheffer, H. , Brunt, E. R. , Ippel, P. F. , … Kremer, H. (2002). Spinocerebellar ataxias in the Netherlands: Prevalence and age at onset variance analysis. Neurology, 58(5), 702–708. 10.1212/wnl.58.5.702 11889231

[mgg31314-bib-0030] Wang, C. , Peng, H. , Li, J. , Ding, D. , Chen, Z. , Long, Z. , … Jiang, H. (2017). Alteration of methylation status in the ATXN3 gene promoter region is linked to the SCA3/MJD. Neurobiology of Aging, 53, 192–195. 10.1016/j.neurobiolaging.2016.12.014 28094059

[mgg31314-bib-0031] Zeng, S. , Zeng, J. , He, M. , Zeng, X. , Zhou, Y. , Liu, Z. , … Wang, J. (2015). Chinese homozygous Machado‐Joseph disease (MJD)/SCA3: A case report. Journal of Human Genetics, 60(3), 157–160. 10.1038/jhg.2014.117 25566755

[mgg31314-bib-0032] Zhou, Q. , Ni, W. , Dong, Y. , Wang, N. , Gan, S. R. , & Wu, Z. Y. (2014). The role of apolipoprotein E as a risk factor for an earlier age at onset for Machado‐Joseph disease is doubtful. PLoS ONE, 9(11), e111356 10.1371/journal.pone.0111356 25369462 PMC4219713

[mgg31314-bib-0033] Zühlke, C. H. , Spranger, M. , Spranger, S. , Voigt, R. , Lanz, M. , Gehlken, U. , … Schwinger, E. (2003). SCA17 caused by homozygous repeat expansion in TBP due to partial isodisomy 6. European Journal of Human Genetics, 11(8), 629–632. 10.1038/sj.ejhg.5201018 12891385

